# Strengthening Public Health Scholarship in Sudan: The Role of Leadership and Mentorship Development

**DOI:** 10.4269/ajtmh.22-0377

**Published:** 2022-11-07

**Authors:** Nükte Göç, Saria Hassan, Ibrahim Bani, Suad Babiker, Mahmoud Hilali, Zeinab Ibrahim, Arwa Gaddal, Linda Saleh, Eman Mukhtar Salih, Mayur M. Desai, Erika Linnander

**Affiliations:** ^1^ Global Health Leadership Initiative, Yale School of Public Health, New Haven, Connecticut;; ^2^ Rollins School of Public Health, Emory University, Atlanta, Georgia;; ^3^ School of Medicine, Emory University, Atlanta, Georgia;; ^4^ College of Medicine, Ajman University, Ajman, United Arab Emirates;; ^5^ School of Medicine, Ahfad University, Omdurman, Sudan;; ^6^ Blue Nile National Institute for Communicable Diseases, University of Gezira, Wad Madani, Sudan;; ^7^ School of Medicine, University of Khartoum, Khartoum, Sudan;; ^8^ School of Medicine, Alneelain University, Khartoum, Sudan;; ^9^ Department of Teaching and Learning, Public Health Institute of Sudan, Khartoum, Sudan;; ^10^ Directorate General of Global Health, Sudan Federal Ministry of Health, Khartoum, Sudan;; ^11^ Department of Chronic Disease Epidemiology, Yale School of Public Health, New Haven, Connecticut;; ^12^ Department of Health Policy and Management, Yale School of Public Health, New Haven, Connecticut

## Abstract

A robust public health workforce in Sudan is essential for accelerating progress toward the Sustainable Development Goals, and strengthening public health education is a priority for the Ministries of Health and Higher Education. Faculty at public health training institutions are a critical resource. Globally, development programs for junior to midlevel public health faculty have been well documented. However, most involved direct partnership between a university from the Global North and only one or two universities from the Global South, only one included an explicit focus on creation of a leadership network, and none were launched as fully virtual collaborations. Therefore, we conducted a mixed-method evaluation of the fully virtual Yale–Sudan Program for Research Leadership in Public Health. We used program records, participant feedback, competency assessment, and network analysis to evaluate 1) participant engagement, 2) change in skill, and 3) change in collaboration. The program achieved a 93% graduation rate. All participants would “definitely” recommend the program and described the live virtual sessions as engaging, effective, and accessible. We observed progress toward learning objectives and significant increases in 13 of 14 leadership and mentorship competency domains. Collaboration across Sudanese institutions increased, including an almost doubling in the number of pairs reporting scholarly collaboration. Eight authorship teams are actively working toward peer-reviewed publications. The program engaged scholars and policymakers from across Sudan and the Sudanese diaspora achieved high levels of co-creation and continues despite significant political unrest in the country, serving as a promising model for strengthening of public health education in Sudan.

## INTRODUCTION

Sudan is the third most populous country in Africa (40 million population)^1^ and ranks among the bottom 10% of countries in progress toward reaching its 2030 Sustainable Development Goals.^2^ Given the significant morbidity and mortality associated with this limited progress, there is an urgent need to address multiple levels of public health in Sudan. A robust public health workforce is an essential component in addressing these gaps, and strengthening public health education in Sudan has been identified as a priority by Sudan’s Ministers of Health and Higher Education.

In response, several of Sudan’s leading educational institutions (Ahfad University for Women, University of Gezira, University of Khartoum, Neelain University, and the Public Health Institute of Sudan) came together to identify strategies to strengthen public health education. In each organization, junior faculty were identified as a critical stakeholder group, representing the future of public health scholarship in Sudan and responsible for providing public health education for future generations. Although the specific profiles of these junior faculty vary across institutions, they generally have heavy teaching loads, mentorship, and supervisory responsibilities for students and postgraduate researchers and are working to establish funded research portfolios with little formal mentorship or support. Levels of burnout and turnover are high. Faculty development programs to promote mentorship and foster leadership among junior to midlevel public health faculty have been well documented, including in lower- and middle-income country settings.^3^^,^^4^ However, most of these involved direct partnership between a university from the Global North and only one or two universities from the Global South, only one included an explicit focus on creation of a leadership network,^5^ and none were launched as fully virtual collaborations.

Therefore, we studied the implementation experience and short-term outcomes associated with the Yale–Sudan Program for Research Leadership in Public Health. The inaugural program was designed to foster a network of junior and midlevel faculty with the ability to 1) assume leadership roles within their institutions, 2) serve as more effective mentors to their trainees, and 3) influence scholarship and practice through scientific writing. The partners also acknowledged that training of individual junior faculty members would be insufficient to drive the level of transformation envisioned and committed to fostering organizational systems and dynamics that will allow these rising research leaders to flourish. Therefore, the program included design features and curricular content to 1) foster collaboration between partner institutions in Sudan to capture synergies and leverage the resources of each institution and 2) promote alignment across levels of hierarchy within each institution so that the junior faculty that were at the heart of this program were also well supported by their own mentors. We used a mixed-methods evaluation of the inaugural cohort of the program to assess participant engagement, change in mentorship and leadership competencies, and levels of collaboration. The results are expected to be useful to scholars and practitioners committed to the strengthening of institutes and schools of public health, in leadership development and public health workforce strengthening, and in innovative models for global collaboration.

## METHODS

### Partner organizations.

The partnership network was developed through introductory visits among organizational leadership that were catalyzed by members of the Sudanese diaspora committed to strengthening the public health workforce in Sudan. The partnership network included four of Sudan’s leading universities: Ahfad University for Women, University of Gezira, University of Khartoum, and Neelain University. To promote alignment with national public health priorities and practices, the partnership also engaged Sudan’s Federal Ministry of Health (FMoH) and the Public Health Institute, a branch of the Ministry. The Yale School of Public Health provided capacity-building support. Each partner institution identified a coordinator to support communication, contextualize the program, and align with current events in each institution and at the national level. The program was funded by a grant to Yale University from the U.S. Embassy in Khartoum.

### Recruitment.

We aimed to engage junior to mid-career full-time faculty who were currently providing mentorship to more junior scholars, postdoctoral trainees, and/or students. We sent official invitation letters to university deans, asking them to nominate up to five junior to mid-career full-time faculty who they believe would benefit from the program. We also highlighted that their selection should be “rising-star” faculty who have been identified as having high potential to take on more senior roles and who are currently providing mentorship to more junior scholars, postdoctoral trainees, or students. Each nominee was asked to submit their resume, application form, and personal statement to complete their application. Twenty-eight participants were identified to join the program. Each participant was also asked to identify a senior-level faculty member to act as their mentor and champion for their growth during the program. This could have been a new or an existing mentorship relationship, and these champions were engaged in select parts of the program.

### Curriculum.

**Figure 1.**
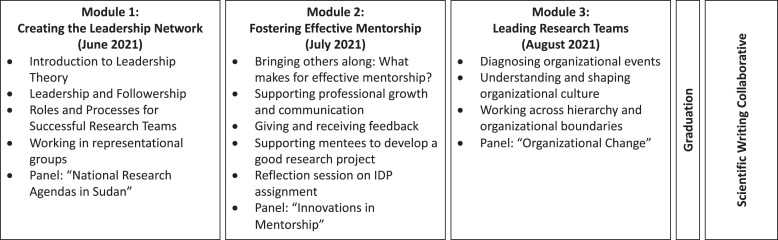
Program overview.

The core curriculum was based on an in-person training program for HIV/AIDS and Tuberculosis Researchers in South Africa, codeveloped with the University of Witwatersrand School of Public Health and Aurum Institute (previously described^5^), adapted for Sudan’s country context and for feasibility during COVID-19. The resulting curriculum was delivered as a series of three, month-long modules (Figure 1). *Creating the Leadership Network* aimed to provide an orientation to the program, build relationships among participants and between participants and their mentors, and prepare participants to elevate their careers in the context of both national research priorities and home institution strategic objectives. *Fostering Effective Mentorship* aimed to equip participants to analyze major theories of mentorship in the context of their own institutions, use approaches and tools to function effectively in mentorship roles, and advocate for their own mentorship and professional development. *Leading Research Teams* aimed to prepare participants to take on increasing research leadership roles in their home organizations, including leading multidisciplinary research teams and in roles of administrative leadership. Of note, each module of the program was adapted through consultation with the program coordinator from each of the Sudanese partner universities and institutes. A detailed description of the sessions included in each of these modules is outlined in Supplemental File 1.

Following completion of the three core modules, graduates were invited to apply to a scientific writing collaborative to support the development of publishable manuscripts that will inform policy and practice. Alumni applied to the collaborative by identifying a paper to develop into a peer-reviewed publication. This could be their own paper or that of a mentee (i.e., an exceptionally promising master’s thesis). After an in-depth application review process, nine successful participants were selected to work with a Yale faculty mentor to build an authorship team, identify a target journal, and work toward submitting the manuscript. The collaborative included a launch webinar on practical approaches and best practices for scientific writing, access to an online repository of resources on best practices in scientific writing, self-paced work within mentor–mentee teams, and virtual check-ins via WhatsApp to promote accountability and peer support.

### Format.

The program was delivered as a fully virtual collaboration in the context of COVID-19. To overcome expected challenges associated with the virtual format (bandwidth and connectivity challenges as well as potentially limited relationship-building opportunities), we invested in the following: 1) a structured risk assessment with participants to understand and align to their work life (whether they work remote, in-person, or hybrid), time, and day preferences to ensure their connectivity, preferred way of communication, and workload; 2) a repository of program materials (including live session recordings) for asynchronous access; 3) use of a WhatsApp group chat to foster connection and engagement; and (4) weekly meetings with program coordinators from each site to update on changes in the local contexts as well as to promote troubleshooting and follow-up with individual participants as needed.

The blended learning model included asynchronous content on essential capacities and practices, synchronous sessions to foster understanding and integration, and individual- and team-based assignments to reinforce learning and foster systems change. Each module included a set of asynchronous learning materials (readings, videos) delivered via the Yale School of Public Health Canvas platform, one live session per week delivered via Zoom^®^ and supported by small breakout room facilitation in Miro^®^, and individual assignments to promote integration and application in participants’ home organization. Each module culminated in a panel discussion that engaged leadership from Sudan and the Sudanese diaspora. Participants committed to approximately 4 to 5 hours of coursework per week. All session recordings and related materials were made available in a program archive on Canvas^®^ and in Box^®^ for future use by participants and replication/adaptation by partner institutions.

Participants who engaged in at least 85% of the live Zoom sessions (or accessed the recordings), completed assignments related to creation and use of Individual Development Plans with their mentor/champion and completed the competency self-assessments at the beginning and end of the program earned a certificate of completion from the Yale School of Public Health and their home institution.

### Evaluation methods.

We used a mixed-methods design for the evaluation. Specifically, a mixed-method design integrates qualitative and quantitative methods that are complementary in their strengths and liabilities to measure interrelated aspects of implementation, generating a rich understanding of program implementation and allowing for expansion beyond any single data source to understand the impact of the program.^6^^,^^7^ Our evaluation drew on four data sources. First, we used program administration data to assess participant engagement. Second, we used an anonymous, voluntary online survey after each module to understand participant satisfaction with program content and logistics, progress toward learning objectives, and recommendations for improvement. Participants rated their satisfaction for each component through a 5-point Likert scale and also provided written feedback to the open-ended questions asking about what worked well and what could be improved about the program (see Supplemental File 2).

Third, at launch and graduation, participants completed a confidential online self-assessment survey of their mentorship and leadership competencies. Longitudinal changes in mentorship competencies were assessed using the validated 26-item Mentorship Competency Assessment (MCA).^8^ The 26 skills in the MCA are categorized into six domains as follows: 1) maintaining effective communication, 2) aligning expectations, 3) assessing understanding, 4) fostering independence, 5) addressing diversity, and 6) promoting professional development. Responses are scored using a 7-point Likert scale from “not at all skilled” to “extremely skilled.” We used paired *t* tests to compare changes from the program’s start to the end in average scores for each skill and for the six summary competencies. Similarly, longitudinal changes in leadership competencies were assessed using the Leadership Competency Assessment (LCA) tool.^9^ The 52 skills in the LCA are categorized into eight domains: 1) system thinking, 2) political advocacy, 3) interdisciplinary collaboration, 4) effective communication, 5) change management, 6) emotional intelligence, 7) organizational development, and 8) ethics and social responsibility (see Supplemental File 3 for the 26 mentorship and 52 leadership skills assessed). We again used paired *t* tests to evaluate pre–post change in each domain.

Fourth, participants completed a voluntary, confidential network survey at the start of the program and again at the end of the program. Each participant was asked whether they collaborated with the other participants in the following ways: 1) sharing research in progress, 2) sharing information about funding opportunities, 3) cosponsoring activities (programs/advocacy), 4) financial relationships/contracts, 5) sharing data, 6) serving on professional committees together, and 7) other. Using UCINET software,^10^ we assessed changes in the numbers of connections each participant had with others in their institution and across institutions.

Information from the four data sources was integrated based on the Kirkpatrick Model, a widely recognized model to understand the results of educational programs at multiple levels from learner reaction to organizational impact^11^ and characterize the impact of the program at three levels: 1) learner engagement and satisfaction with the program, 2) the degree to which learners acquire the intended competencies, and 3) the impact of the program on the density of the research leadership network across the partner institutions.

This evaluation was deemed exempt from continuing review by the Yale Institutional Review Board.

## RESULTS

### Participants.

Following the recruitment process, 28 participants from partner institutions enrolled in the program (Ahfad University for Women: five, University of Gezira: five, University of Khartoum: six, Neelain University: four, and the Public Health Institute of Sudan: five, Federal Ministry of Health: three). In terms of gender, 75% of the participants were women, and 25% were men. Table 1 outlines the participant characteristics.

**Table 1:** Participant characteristics (*N* = 28)

Characteristic	*n*	(%)
Gender		
Female	21	(75%)
Male	7	(25%)
Institution		
Ahfad University for Women	5	(18%)
University of Gezira	5	(18%)
University of Khartoum	6	(21%)
Neelain University	4	(14%)
Public Health Institute	5	(18%)
Federal Ministry of Health	3	(11%)

### Participant engagement.

In total, 26 of 28 participants (93%) successfully completed the program. The average attendance at each live session was 70%. The panel discussions at the end of each module were a highlight of the program, with 100% of the participants either live-streaming or watching the recording of the panel sessions, and many requesting to share the recordings across their professional networks.

After each of the three modules, 100% of program participants stated they would “definitely recommend” the program to peers. Participants also expressed strong satisfaction with session logistics in terms of Zoom breakout room management, live session facilitation, Canvas materials and access, and program communication including initial meetings, receiving schedules, and programmatic updates. Table 2 outlines participant feedback on program logistics, in terms of Zoom breakout room management, live session facilitation, Canvas materials and access, and program communication including initial meetings, receiving schedules, and programmatic updates.

**Table 2:** Participant feedback on program logistics

Program component	% Highly satisfied
Live session facilitation	94%
Breakout rooms	91%
Canvas access and materials	88%
Logistics and communications	87%

Participants described the live virtual sessions as engaging, effective, and accessible. Similar to our experiences in adapting other leadership development modules to fully remote delivery in the context of COVID-19, this approach fostered integration of didactic and applied work, and rapid translation of new skills into practice.

All sessions worked well except for connection and electricity problems; on the other hand live sessions and breakout rooms were just **perfect and welcoming**.

The excellent organization of the program and the **positive atmosphere** that Yale staff has created to encourage participants to contribute to the sessions has been accepting of different cultures and different levels of abilities.

The program is well structured and professionally directed. Live sessions are the best part because of the excellent presenters and the active thoughtful input from participants. For me it has been [the] sense I have the chance to discuss various core concepts in **such good mixture of theory and practicality.**

An effective online communication was demonstrated through the interaction and engagement of the instructors with the students. To my belief, the teaching and learning process adopted in the program was well planned and delivered. Thus, it enabled the program to successfully overcome the difficulties typically faced in virtual classes. **This experience has supported the extension of my learning opportunities, beyond the traditional scope of face-to-face learning.** We were challenged to critically analyze our work situations and context, with every discussion and reflection in class.

### Participant learning.

In the exit surveys after each module, participants reported high levels of understanding toward learning objectives. Between 74% and 100% of participants met each of 11 learning objectives. Table 3 outlines participant’s level of understanding toward learning objectives as reported in the exit surveys.

**Table 3:** Percentage of participants who reported meeting learning objectives

Learning objectives	% Agree or strongly agree
Module 1 (*N* = 23)	
I understand leadership theories and their implications for research teams.	87%
I understand the relationship between leadership and followership and feel equipped to be an effective leader and follower.	96%
I believe that I have an understanding of mechanisms to promote role clarity, accountability, development of productive conflict, and individual and group decision-making processes.	74%
I understand the advantages of working in representational groups for scientific productivity and development of practical approaches to mitigating the inherent challenges.	91%
Module 2 (*N* = 24)	
I understand the concept of mentorship in research, including goals and attributes of mentoring relationships, challenges to mentorship, mentorship networks, and sponsorship.	100%
I understand the skills and tools needed to support mentorship, including effective communication, supporting professional growth, and coaching on individual development plans.	88%
I have the skills needed to help students/trainees properly scope a research project and present tools to aid in developing a good project.	96%
I can address mentorship challenges including initiating a difficult conversation, promoting psychological safety, and raising sensitive issues.	83%
Module 3 (*N* = 26)	
I have the skills needed to understand and address organizational dynamics using multiple levels of analysis (individual, interpersonal, group, and intergroup).	89%
I have the skills needed to collaborate across group boundaries.	97%
I have the skills needed to promote positive shifts in organizational culture.	100%

Participants also offered descriptions of early application of their learning.

I realized there are some people who come to discuss with me their career and other issues constantly and I do the same with other. Now I realize this can be more efficient, effective, and structured.

I’m interested in organizational dynamics due to my current position in our staff trade union, so understanding and shaping organizational culture were the best to learn about; opening my eyes to new approaches to introduce change.

**Figure 2.**
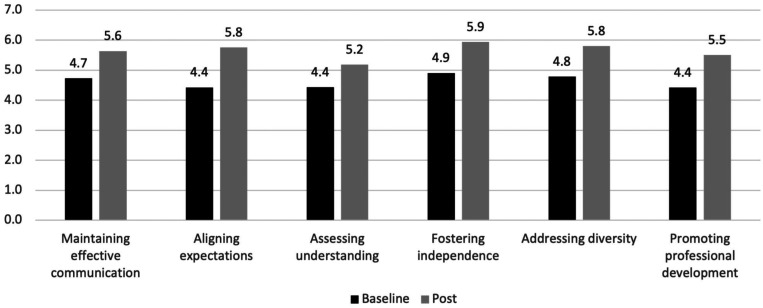
Change in mentorship competencies (*N* = 23).

Participants showed statistically significant (*P* < 0.05) increases in five of the six domains of the MCA: maintaining effective communication, aligning expectations, fostering independence, addressing diversity, and promoting professional development. Consistent with the curricular focus on the development and use of Individual Development Plans, participants showed the greatest pre–post increase on the aligning expectations domain (1.4-point increase), followed by promoting professional development (1.1-point increase), fostering independence (1.0-point increase), and addressing diversity (1.0-point increase). Figure 2 shows the level of change in pre- and post-program mentorship competencies.

**Figure 3.**
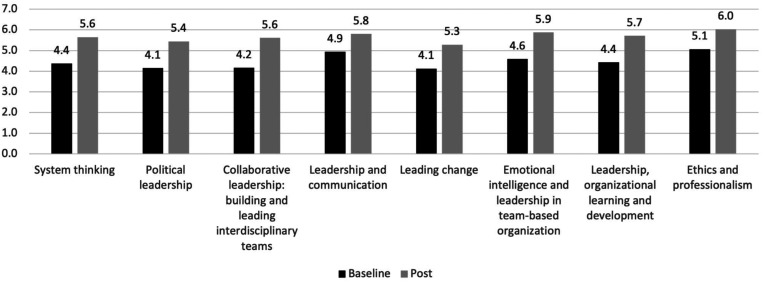
Change in leadership competencies (*N* = 23).

Participants also showed statistically significant (*P* < 0.05) increases in each leadership domain, including 1) systems thinking, 2) political leadership, 3) collaborative leadership: building and leading interdisciplinary teams, 4) leadership and communication, 5) leading change, 6) emotional intelligence and leadership in team-based organization, 7) leadership, organizational learning and development, and 8) ethics and professionalism. Consistent with the program curriculum, participants reported the highest increase in collaborative leadership: building and leading interdisciplinary teams (1.4-point increase), followed by 1.3-point increases in political leadership; emotional intelligence and leadership in team-based organization; and leadership, organizational learning and development. Figure 3 shows the level of change in pre- and post-program leadership competencies.

### Change in the research leadership network.

**Figure 4.**
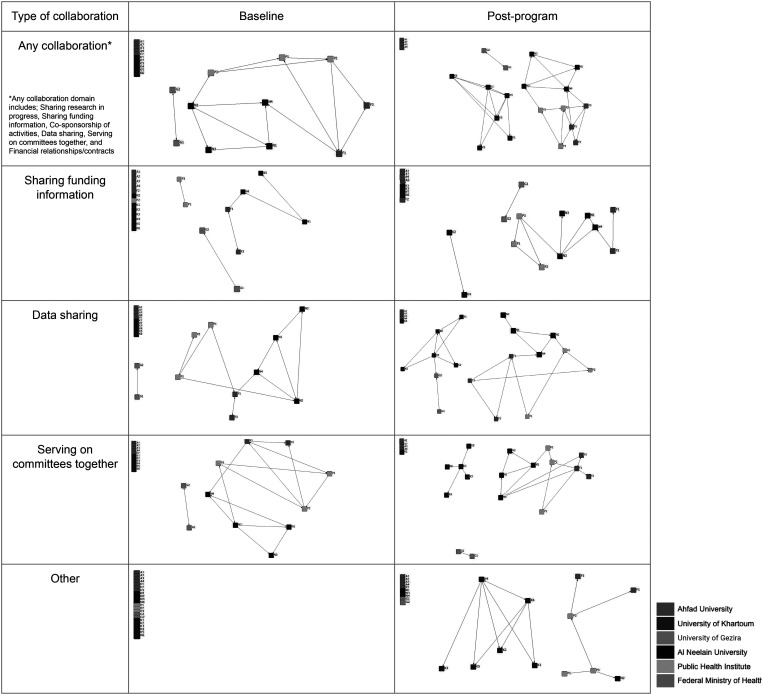
Network connections before and after the program (*N* = 22). Nodes represent individual participants, with shading to indicate their home institution. Lines represent reported collaboration between participants.

The level of collaboration within and between institutions increased during the project period (Figure 4). The number of pairs of individuals who engaged in any form of collaboration increased from 15 to 28. This increase was driven by pairs who reported sharing funding information (up from 6 to 11 pairs), sharing data (up from 13 to 20 pairs), and serving on committees together (up from 13 to 18 pairs). We also observed 13 new collaborations focusing on institutionalizing mentorship and e-learning (“other” in Figure 4). Of note, University of Khartoum participants had shown no interaction before the program started but collaborated within every domain except cosponsorship activities and financial relationships and contracts by the end of the program. Figure 4 shows level of collaboration within and between institutions increased during the project period.

## DISCUSSION

The inaugural Yale–Sudan Program for Research Leadership in Public Health was successfully delivered in a fully virtual format with participants from across six flagship public health institutions in Sudan. The program achieved high levels of participant engagement (93% graduation rate; 100% would “definitely recommend” to their peers); significant impact on mentorship and leadership competencies; and measurable strengthening of research collaborations within and across partner institutions. As part of the scientific writing collaborative, eight authorship teams are actively working toward producing peer-reviewed publications.

Most notably, the partners overcame barriers expected of a fully virtual program in Sudan (telecommunications barriers and relationship-building challenges) to create a program that was experienced as engaging, effective, and accessible. Further, although the program was launched during a period of relative political stability, active collaborations across institutions have persisted despite the onset of a period of sustained political unrest associated with military rule starting in October 2021.

Our results are comparable to those from a similar program for researchers in South Africa (previously described^5^). The program completion rate in South Africa and Sudan were comparable (88% and 93%, respectively), and satisfaction with the program was higher in Sudan (100% would recommend, compared with 75% in South Africa). Participants from the two programs showed similar gains in mentorship competencies and in levels of collaboration, despite the anticipated disadvantages of the fully virtual format.

The curriculum for this current program built on two specific lessons learned from the South African experience. First, one of the recommendations from the South Africa experience was to build an explicit mentor–mentee dyad into the design of the program. The design in Sudan incorporated this recommendation (called “champion” and “participant”), and we achieved high levels of engagement by the champions in live sessions and assignments. Second, in both programs, national partners advocated for the inclusion of panel sessions at the end of each module to contextualize the program content and encourage conversations with leading national scholars and practitioners. Public health and medical professionals from the diaspora have been described as an underused resource for low- and middle-income country health systems strengthening efforts,^12^ and the current program design aimed to better leverage this expertise and enthusiasm of the Sudanese diaspora. Although participants’ attendance at the panel sessions was optional, 100% of the participants in Sudan either joined the live virtual sessions or watched the recording, and many requested to share the recordings across their professional networks, highlighting the perceived value of this curricular component.

Our results should be interpreted in light of several limitations. First, assessment of progress toward program objectives and changes in competencies are based on participants’ self-report, and not corroborated by inputs from supervisors or peers. However, self-reported assessments are a widely used approach, and the results of the LCA, MCA, and network analyses indicate comparable results with previous studies with a similar outcome evaluation setting.^5^^,^^13^ Second, follow-up study is needed to understand the extent to which the observed competency improvements are sustained over time. However, the program design included specific attention to the creation of structures and relationships that are expected to bolster capacity within each institution beyond the competency gains of any individual participant. Longer-term study would also allow for evaluation of the impact of the program on the career trajectories of alumni and the strategic goal of retention of junior and midlevel faculty in each institution. Lastly, the future state of the program is unclear due to political unrest and competing priorities in the country. However, we demonstrated measurable impact in a short amount of time, and results presented in this article provide promising evidence for sustained collaboration with our partner institutions in Sudan.

## CONCLUSION

The Yale–Sudan Program for Research Leadership in Public Health engaged scholars and policymakers from across Sudan and the Sudanese diaspora, achieved high levels of co-creation with local coordinators, and continues despite significant political unrest in the country, serving as a promising model for continued partnership toward strengthening of the public health workforce in Sudan and other country settings. We expect that our results will be useful to scholars and practitioners committed to the strengthening of institutes and schools of public health, in leadership development and public health workforce strengthening, and in innovative models for global collaboration.

## Supplemental files


Supplemental materials
