# Supplementary material for: Strengthening Public Health Scholarship in Sudan: The Role of Leadership and Mentorship Development
Source: Am J Trop Med Hyg. 2022 Nov 7;107(6):1323–30. doi: 10.4269/ajtmh.22-0377 (PMC9768261; doi:10.4269/ajtmh.22-0377)
Supplement: Supplementary file 1 [file tpmd220377.SD1.pdf]

**Supplemental File 1: Participant Feedback Survey**

Thank you for your participation in Yale-Sudan Program Research Leadership in Public Health. Please take a moment to provide some feedback on your experience. This will help us ensure your satisfaction and further improvements of the program. Your responses are entirely confidential.

| **To what extent do you agree with the following statements?** | | | | |
|---|---|---|---|---|
| | Strongly Agree | Agree | Disagree | Strongly disagree |
| I have understood leadership theories and its implications for the research teams. | | | | |
| I understand the relationship between leadership and followership and feel equipped to be an effective a leader and a follower. | | | | |
| I believe that I have an understanding of mechanisms to promote role clarity, accountability, development of productive conflict, and individual and group decision-making processes. | | | | |
| I understand the advantages of working in representational groups for scientific productivity and development of practical approaches to mitigating the inherent challenges. | | | | |

| **How satisfied were you with each of the following program elements?** | | | |
|---|---|---|---|
| | Very Satisfied | Somewhat Satisfied | Not at all Satisfied |
| Orientation to program | | | |
| Pre-Program Logistics Communication | | | |
| Session schedule | | | |
| Canvas access and materials | | | |
| Breakout rooms | | | |
| Live session facilitation | | | |
| Panel session | | | |

| **What could be improved about the program?** |
| --- |
| |
| **What works well in the program so far?** |
| |

| Would you recommend Yale-Sudan Program to your peers from another country? | | | | |
| --- | --- | --- | --- | --- |
| ☐ Yes, definitely | ☐ Maybe | ☐ Not likely | ☐ No, definitely not | ☐ No opinion |
| Think about your experience in Yale-Sudan Program so far (including enrollment, online modules, live sessions, panel session, breakout rooms, communication). So far, what has worked well? | | | | |
| | | | | |
| Think about your experience in Yale-Sudan Program so far including enrollment, online modules, live sessions, panel session, breakout rooms, communication). So far, what has not worked well? What would you change? | | | | |
| | | | | |
| Please use this space to provide any additional feedback or suggestions. | | | | |
| | | | | |

**Supplemental File 2: Mentorship and Leadership Competencies**

| **Mentorship** | |
|---|---|
| Maintaining effective communication | 1. Active listening |
| | 2. Providing constructive feedback |
| | 3. Establishing a relationship based on trust |
| | 4. Identifying and accommodating different communication styles |
| | 5. Employing strategies to improve communication with mentees |
| | 6. Coordinating effectively with your mentees' other mentors |
| Aligning expectations | 7. Working with mentees to set clear expectations of the mentoring relationship |
| | 8. Aligning your expectations with your mentees' |
| | 9. Considering how personal and professional differences may impact expectations |
| | 10. Working with mentees to set research goals |
| | 11. Helping mentees develop strategies to meet goals |
| Assessing understanding | 12. Accurately estimating your mentees' level of scientific knowledge |
| | 13. Accurately estimating your mentees' ability to conduct research |
| | 14. Employing strategies to enhance your mentees' knowledge and abilities |
| Fostering independence | 15. Motivating your mentees |
| | 16. Building mentees' confidence |
| | 17. Stimulating your mentees' creativity |
| | 18. Acknowledging your mentees' professional contributions |
| | 19. Negotiating a path to professional independence with your mentees |
| Addressing diversity | 20. Taking into account the biases and prejudices you bring to the mentor/mentee relationship |
| | 21. Working effectively with mentees whose personal background is different from your own (age, race, gender, class, culture, religion, family composition) |
| Promoting professional development | 22. Helping your mentees network effectively |
| | 23. Helping your mentees set career goals |
| | 24. Helping your mentees balance work with their personal life |
| | 25. Understanding your impact as a role model |
| | 26. Helping your mentees acquire resources (e.g. grants, etc.) |

| Leadership | |
|---|---|
| System thinking | 1. Understand current public health issues and engage in systemic change to address them. |
| | 2. Synthesize and integrate divergent viewpoints for the good of an organization. |
| | 5. Recognize the relevance of adaptive leadership and use it in the appropriate circumstances. |
| | 6. Recognize the relevance of leading from behind and use this in the appropriate circumstances. |
| | 7. Identify opportunities for growth, innovation, change and development of the organization. |

| | |
|---|---|
| Political advocacy | 8. Foresee potential impacts and consequences of decision-making in both internal and external situations. |
| | 9. Understand and apply effective techniques for working with boards and governance structures. |
| | 11. Promote the regional and national public health agenda. |
| | 12. Translate broad strategies into practical terms for others. |
| | 13. Build alliances, partnerships, and coalitions to improve the health of the community or population being served. |
| | 14. Identify and engage stakeholders in interdisciplinary projects to improve public health. |
| | 15. Advocate and participate in public health policy initiatives at the local, national and/or international levels. |
| Interdisciplinary collaboration | 16. Provide an environment conductive to opinion sharing. |
| | 17. Model effective group process behaviours including listening, dialoguing, negotiating, rewarding, encouraging, and motivating. |
| | 19. Understand and manage expectations. |
| | 20. Offer opportunities for collaborative learning and quality improvement. |
| Effective communication | 25. Share views in a non-judgmental, non-threatening way. |
| | 26. Exercise the sensitivity needed to communicate with diverse cultures and disciplines. |
| | 27. Effectively use negotiation skills to mediate disputes and find appropriate and workable solutions. |
| Change management | 29. Manage staff to effectively deal with change. |
| | 30. Serve as a driving force for change, including strategies of change. |
| | 31. Make strategic decisions based on recognized values, priorities and resources. |
| | 32. Identify and communicate new system structures as needs are identified and opportunity arises. |
| | 33. Ensure that organizational practices are aligned with changes in the public health system and the larger social, political, and economic environment. |
| Emotional intelligence | 34. Demonstrate awareness of the impact of your own beliefs, values, and behaviours on your own decision-making and the reactions of others. |
| | 35. Demonstrate empathy and concern for people as individuals while ensuring that organizational goals and objectives are met. |
| | 36. Be aware of the impact of your own behaviors and reactions on the behaviors and reactions of others. |
| | 37. Demonstrate personal responsibility and accountability for the achievement of a given task. |
| | 38. Respond appropriately to the positive criticism of others about your own behavior or performance. |
| Organizational development | 40. Foster an environment of trust. |
| | 42. Advocate for learning opportunities within the organization. |
| | 43. Create and communicate a shared vision for the future and inspire team members to achieve it. |
| | 44. Encourage others to feel ownership in the public health mission in the organization. |
| | 45. Assist others to clarify thinking, create consensus, and develop ideas into |

| | |
|---|---|
| | actionable plans. |
| | 46. Offer opportunities for collaborative learning and quality improvement. |
| Ethics and social responsibility | 47. Adhere to ethical legal and regulatory standards. |
| | 50. Respect diverse cultures and build upon the strength of diversity to bring about innovation and added value in the work environment. |
| | 51. Practice and promote professional accountability and social responsibility. |
| | 52. Actively work towards reducing inequalities in access to public health. |

**Supplemental Table 1. Curriculum**

| **Module 1: Creating the Leadership Network (June, 2021)**<br>The goal of this module was to provide an orientation to the program, build relationships among participants and between participants and their mentors, and prepare participants to elevate their careers in the context of both national research priorities and home institution strategic objectives. | |
|---|---|
| **Session 1**: Introduction to Leadership Theory | We described the evolution of leadership theories over time, with emphasis on the implications for the way we model and develop leadership within research teams. |
| **Session 2:** Leadership and Followership | We explored the relationship between leadership and followership, with attention to common misperceptions that hinder organizational effectiveness and identification of concrete actions that promote effective followership. |
| **Session 2**: Roles and Processes for Successful Research Teams | Building on our understanding of leadership as a role, we identified additional roles and processes required for successful research collaborations, including mechanisms to promote role clarity, accountability, development of productive conflict, and individual and group decision-making processes. |
| **Session 3:** Working in representational groups | We used an experiential learning simulation to highlight the challenges of working with representational groups, followed by (1) discussion of the advantages to working in representational groups for scientific productivity and (2) development of practical approaches to mitigating the inherent challenges. |
| **Session 4**: Working across hierarchy and organizational boundaries | We used an experiential learning exercise to identify boundaries among participants' groups and promote reflection within and between groups, with the goal of identifying and addressing misperceptions and promoting empathy as a foundation for collaboration. |
| **First Panel Session** | We curated panel session titled "National Research Agenda in Sudan", moderated by Professor Ibrahim Bani. We welcomed panelists including, Professor Ahmed Hassan Fahal, Professor Mustafa A. Salih, Professor Nafisa M. Bedri, and Professor Taha E. Taha. |

| **Module 2: Fostering Effective Mentorship (July, 2021)**<br>The goal of this module is to equip participants to analyze major theories of mentorship in the context of their own institutions, employ approaches and tools to function effectively in mentorship roles, and advocate for their own mentorship and professional development. | |
|---|---|
| **Session 1:** Bringing others along: What makes for effective mentorship? | We provided an overview of mentorship in research, including goals and attributes of mentoring relationships, challenges to mentorship, mentorship networks, and sponsorship. The session also included a discussion of current mentorship activities and institutional supports at each of the partner institutions. |
| **Session 2**: Supporting professional growth and communication | We described key skills and tools needed to support mentorship, including effective communication, supporting professional growth, and coaching on individual development plans. |
| **Session 3:** Giving and receiving feedback | We discussed challenges to open communication, preparing for and handling difficult conversations, creating psychological safety, and raising |

| | |
|---|---|
| | sensitive issues. |
| **Session 4:** Supporting Mentees to Develop a Good Research Project | We described the need to help students/trainees properly scope a research project and present tools (FINGER and PICOT) to aid in developing a good project. |
| **Session 5:** Reflection Session on IDP Assignment | Participants were expected to engage in and reflect on their individual development plans to practice and reinforce the topics covered in the sessions. |
| **Second Panel Session** | We curated panel session titled "Innovations in Mentorship", moderated by Professor Ibrahim Bani. We welcomed panelists including, Dr. Abdelrahim Mutwakel Ballal, Dr. Hassan Naser A Mashbari, Dr. Isameldin Abdelbagi Mohamed Abdelbagi, and Dr. Nada A. Fadul. |

| **Module 3: Leading Research Teams (August, 2021)**<br>The goal of this module is to prepare participants to take on increasing research leadership roles in their home organizations, including leading multidisciplinary research teams and in roles of administrative leadership. | |
|---|---|
| **Session 1**: Diagnosing organizational events | Using case examples, we explored commonly frustrating and ineffective organizational dynamics using multiple levels of analysis (individual, interpersonal, group, and intergroup), with implications for the most appropriate intervention/treatment. |
| **Session 2**: Understanding and shaping organizational culture | We focused on relevance for cross-disciplinary and cross-organization research collaborations. Topics in this session included what is organizational culture, why does it matter, and what do the most effective teams do to promote positive shifts in organizational culture? |
| **Session 3:** Working Across Hierarchy and Organizational Boundaries | We used an experiential learning model to identify boundaries and promote reflection within and between groups, with the goal of strengthening collaboration. The session included topics on organizational structures that support or impede collaboration, incentives and disincentives, and understanding and leveraging diverse goals. |
| **Third Panel Session** | We curated panel session titled "Organizational Change", moderated by Professor Ibrahim Bani. We welcomed panelists including, Professor Khaled Al Surmi, Dr. Mayada Abu Affan, Professor AbdelMoniem Sahal Elmardi, Professor Allam Ahmed. |
